# First Evidence of Dinosaurian Secondary Cartilage in the Post-Hatching Skull of *Hypacrosaurus stebingeri* (Dinosauria, Ornithischia)

**DOI:** 10.1371/journal.pone.0036112

**Published:** 2012-04-30

**Authors:** Alida M. Bailleul, Brian K. Hall, John R. Horner

**Affiliations:** 1 Museum of the Rockies, Montana State University, Bozeman, Montana, United States of America; 2 Department of Earth Sciences, Montana State University, Bozeman, Montana, United States of America; 3 Department of Biology, Dalhousie University, Halifax, Nova Scotia, Canada; University of Pennsylvania, United States of America

## Abstract

Bone and calcified cartilage can be fossilized and preserved for hundreds of millions of years. While primary cartilage is fairly well studied in extant and fossilized organisms, nothing is known about secondary cartilage in fossils. In extant birds, secondary cartilage arises after bone formation during embryonic life at articulations, sutures and muscular attachments in order to accommodate mechanical stress. Considering the phylogenetic inclusion of birds within the Dinosauria, we hypothesized a dinosaurian origin for this “avian” tissue. Therefore, histological thin sectioning was used to investigate secondary chondrogenesis in disarticulated craniofacial elements of several post-hatching specimens of the non-avian dinosaur *Hypacrosaurus stebingeri* (Ornithischia, Lambeosaurinae). Secondary cartilage was found on three membrane bones directly involved with masticatory function: (1) as nodules on the dorso-caudal face of a surangular; and (2) on the bucco-caudal face of a maxilla; and (3) between teeth as islets in the alveolar processes of a dentary. Secondary chondrogenesis at these sites is consistent with the locations of secondary cartilage in extant birds and with the induction of the cartilage by different mechanical factors - stress generated by the articulation of the quadrate, stress of a ligamentous or muscular insertion, and stress of tooth formation. Thus, our study reveals the first evidence of “avian” secondary cartilage in a non-avian dinosaur. It pushes the origin of this “avian” tissue deep into dinosaurian ancestry, suggesting the creation of the more appropriate term “dinosaurian” secondary cartilage.

## Introduction

**Figure 1:**
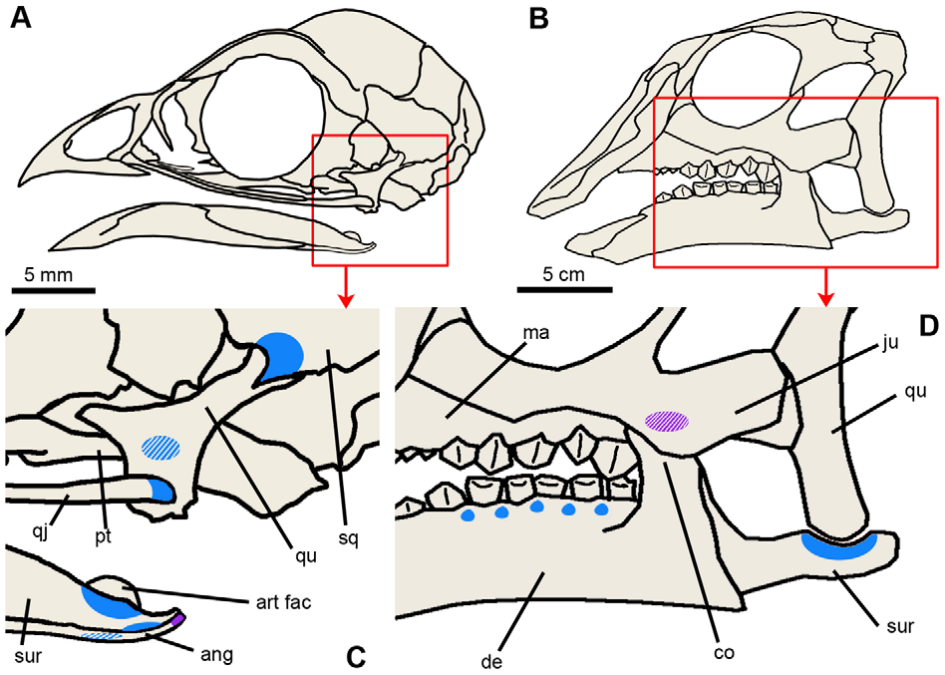
Head skeleton and distribution of secondary cartilage in a newly-hatched chick and a post-hatching *Hypacrosaurus*. (**A**) Skull diagram of a 2 day-old chick *Gallus*. (**B**) Skull diagram of a post-hatching *Hypacrosaurus*. (**C, D**) Detail in the red box in (A) and (B) respectively. Locations of secondary cartilage are indicated in blue (at articulations) and purple (at muscle or ligament insertions). Diagonal lines indicate that secondary cartilage is not located in the first plane of the figure, but more internally (on the lingual faces). In (C), secondary cartilage is found at the following articulations: pterygoid-quadrate, quadratojugal-quadrate, squamosal-quadrate, surangular-angular, surangular-Meckel's cartilage, and angular-Meckel's cartilage (based on [13]). It is also found on the distal tip of the angular at the insertion site of *M. depressor mandibulae* [13]. Note that these sites change during ontogeny, i.e., more and different sites are present in the embryonic chick [18]. In (D), secondary cartilage is found at the surangular-quadrate articulation; on the bucco-caudal face of the maxilla (in contact with the coronoid process of the dentary), and in the alveolar processes of the dentary between teeth. ang, angular; art fac, articular facet of Meckel's cartilage; co, coronoid process; de, dentary; ju, jugal; ma, maxilla; pt, pterygoid; qj, quadratojugal; qu, quadrate; sq, squamosal; sur, surangular.

Like bone microstructure, calcified cartilage can be fossilized and preserved for hundreds of millions of years [1]–[3]. Indeed, primary cartilage has been widely found and documented in fossils (see review [4]). However, another type of calcified cartilage, less studied than primary cartilage and known as secondary cartilage (because it arises after bone formation), has only been described in extant species and never been reported in a fossil so far. In extant birds, “avian” secondary cartilage is found on skull and jawbones and plays an important role in resisting mechanical stress from embryonic development up to adulthood (Figure 1A,C). Considering the phylogenetic inclusion of birds within the Dinosauria [5], we hypothesized a dinosaurian origin for this “avian” tissue. Therefore, we investigated secondary chondrogenesis by means of histological thin sectioning in disarticulated craniofacial elements of several post-hatching specimens of the non-avian dinosaur *Hypacrosaurus stebingeri* (Ornithischia, Lambeosaurinae), from the Upper Cretaceous (Campanian) Two Medicine Formation of Montana [6].

Early ontogenetic stages are the most suitable to study chondrogenesis, therefore these elements are appropriate to our investigation. They represent the youngest non-avian dinosaur skulls ever studied from a histological perspective. A comparison with primary chondrogenesis could be and was undertaken as well. The main aim of the study was to investigate the proposed dinosaurian origin of “avian” secondary cartilage. The presence of this “avian” tissue in a non-avian dinosaur would push its origin deep into the dinosaurian ancestry, and further cement the dinosaurian origin of birds.

### 1. Modes of skeletal formation in vertebrates

Endochondral bones start out with **primary** cartilage. They are located in the postcranium (e.g., limb bones, vertebrae and ribs) and the neurocranium (i.e., the cartilaginous skull). The latter is composed of the cranial base [7] and the sensory capsules (otic, optic and nasal capsules; see [8] for a complete list of endochondral bones in chick skulls). Bone arises (from osteogenic cells brought in by blood vessels) at the center (diaphysis) of the primary cartilaginous models of long bones from which it spreads. In many groups, secondary centers of ossification arise at the ends (epiphyses) of the cartilage models. Cartilage is replaced by bone at growth plates. More precisely, chondrocytes undergo hypertrophy and cellular apoptosis, and vascular invasion brings in osteogenic cells that lay down new bone matrix. This leaves cartilage only at the extremities as the articular cartilage of the joints. The shape of the final bone is laid down in the cartilage model.

Membrane bones, however, ossify directly through the process of intramembranous ossification [7], [9], without a primary cartilaginous model [10], [11]. Membrane bones form the cranial vault (that protects the brain), the face, and the bone(s) of the jaws [12]. The only membrane bones found in the post-cranium are the paired clavicles. Although membrane bones ossify directly without a primary cartilage model, in some species cartilage can arise secondarily, on pre-existing membrane bones, and is therefore called **secondary** cartilage [7], [13], [14].

### 2. “Avian” secondary cartilage

Secondary cartilage arises because of the ability of periosteal cells to respond to mechanical influences by switching their differentiation from osteogenesis to chondrogenesis [15]. The molecular basis of secondary cartilage formation is well known [16]. The organic phase of secondary cartilage presents simultaneously type I and type II collagens, while primary cartilage only secretes type II collagen [7]. In histological sections, secondary cartilage presents a smaller amount of extracellular matrix than primary cartilage [7], but the identification of the former is based essentially and more accurately on its location (i.e., on the articular surface of a pre-existing membrane bone). In addition to this, the organization of the chondrocytes can also give a clue: while primary cartilage is usually organized into long straight tubes at epiphyseal growth plates [17], secondary cartilage lacks this linear organization, with its chondrocytes organized randomly in nodules ([12]; and see the Results section). Being induced and maintained by mechanical influences, secondary cartilages provide important regional adaptive growth [12] and accommodate stress and strain during normal development [15], [18]–[22], but also during fracture repair [22].

In birds, secondary cartilage is present in the growing craniofacial skeleton ([18], [20]–[25] and Figure 1A,C) and the growing clavicle [26]. “Avian” secondary cartilages are initiated (and maintained) as nodules at sites subject to intermittent pressure, such as articulations, sutures and points of insertions of ligaments or masticatory muscles [15]. Secondary cartilages arise during embryonic life and persist after hatching. In adults almost all of the secondary cartilages are resorbed and their place taken by newly formed endochondral bone. The remaining chondrocytes (in the superficial layers) become the chondrocytes of an articular fibrocartilage [20], [21]. This makes embryos and the newly hatched the most suitable stages to study secondary chondrogenesis in birds.

However, secondary cartilage is not unique to birds. It has been widely sought among extant vertebrates and has also been found in two other groups [15]: teleosts and mammals (Text S1). It has not been reported in lissamphibians or in non-avian sauropsids, despite extensive examination of embryos and attempts to induce such cartilage experimentally [27]–[29]. Instead, these animals accommodate stress by forming syndesmoses (i.e., a dense fibrous connective tissue; e.g., see [7], [29]) at the junction of their membrane bones. The most parsimonious interpretation is that the secondary cartilages displayed by the Teleostei, Mammalia, and Aves are not homologous and arose independently [15]. Therefore, within the Archosauria, secondary cartilage (or the dual ability of periosteal cells to form chondro- and osteoblasts) is unique to birds and seems to carry a phylogenetic signal.

## Results

**Figure 2:**
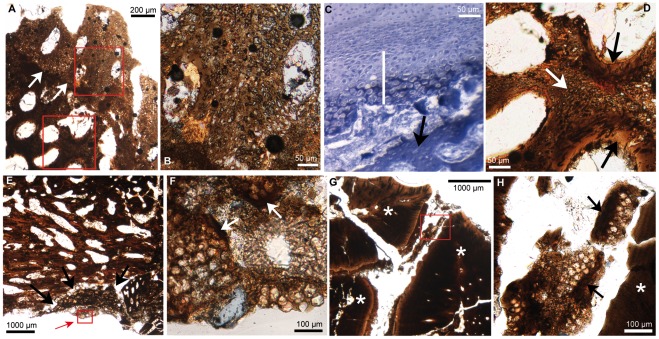
Thin-sections showing secondary cartilage. (**A**) Cross section of the surangular (at the quadrate articulation) of *Hypacrosaurus*. White arrows indicate the limit between bone and secondary cartilage. (**B**) Detail in upper red box in (A). The ovoid lacunae are remnants of hypertrophied chondrocytes. Resorption is evidenced by erosion bays. (**C**) Cross section in a 16 day-old-chick embryo showing Meckel's primary cartilage (uncalcified) above, the perichondrium below it and secondary cartilage (white bar) on eroded surangular bone struts (black arrow). Sudan black B shows that the most mature secondary cartilage is calcified (in dark blue). Adapted from a figure in [40]. (**D**) Detail in lower red box in (A). Secondary cartilage (white arrow) is undergoing resorption and endochondral ossification (black arrows). (**E**) Coronal section in a maxilla. The nodule of secondary cartilage (black arrows) has globular hypertrophied chondrocytes. The area in the small red box (indicated by the red arrow) is detailed in figure (F). (**F**) Detail of red box in (E). The globular and hypertrophied chondrocyte lacunae are encased in a small amount of extracellular matrix. The white arrows indicate bone struts. (**G**) Cross section in the caudal part of a dentary showing teeth (white asterisk). (**H**) Detail of red box in (G). An islet of secondary cartilage is located between a tooth (indicated by the asterisk on the right) and alveolar bone (left). The black arrows show bone struts. Photographs taken under natural light.

Secondary cartilage was found in three locations: on the dorso-caudal face of a surangular at its articulation with the quadrate (Figure 1D, Figure 2A,B,D); on the bucco-caudal face of a maxilla (Figure 1D, Figure 2E,F); and in the alveolar processes of a dentary between teeth (Figure 1D, Figure 2G,H). The surangular and the maxilla each display a nodule of secondary cartilage (Figure 2A,E) while the dentary displays smaller cartilaginous islets (Figure 2G). These cartilages are composed of ovoid lacunae (Figure 2B,D,F,H), interpreted as remnants of hypertrophied cartilage cells separated by a bright mineralized extracellular matrix, the latter appearing darker in the section (Figure 2D). These two nodules and little islets are undergoing endochondral ossification. This is most visible on the surangular where chondroclastic resorption is evidenced by large erosion bays (Figure 2B,D). Subsequent bone apposition is evidenced by bone struts on the walls of some of these erosion bays (Figure 2D). These bone struts are also seen in the nodule of the maxilla (indicated by the white arrows in Figure 2F) and in the islet (indicated by the black arrows in Figure 2H). Secondary cartilages of birds also undergo resorption and endochondral ossification (Figure 2C). We identify unambiguously these cartilages on *Hypacrosaurus* as secondary and not primary for two reasons: (1) they display the typical cellular organization of avian secondary cartilages as described below, an arrangement completely different from the one displayed by primary cartilage; (2) they are located on pre-existing membrane bones and therefore can only have arisen secondarily, after bone formation.

**Figure 3:**
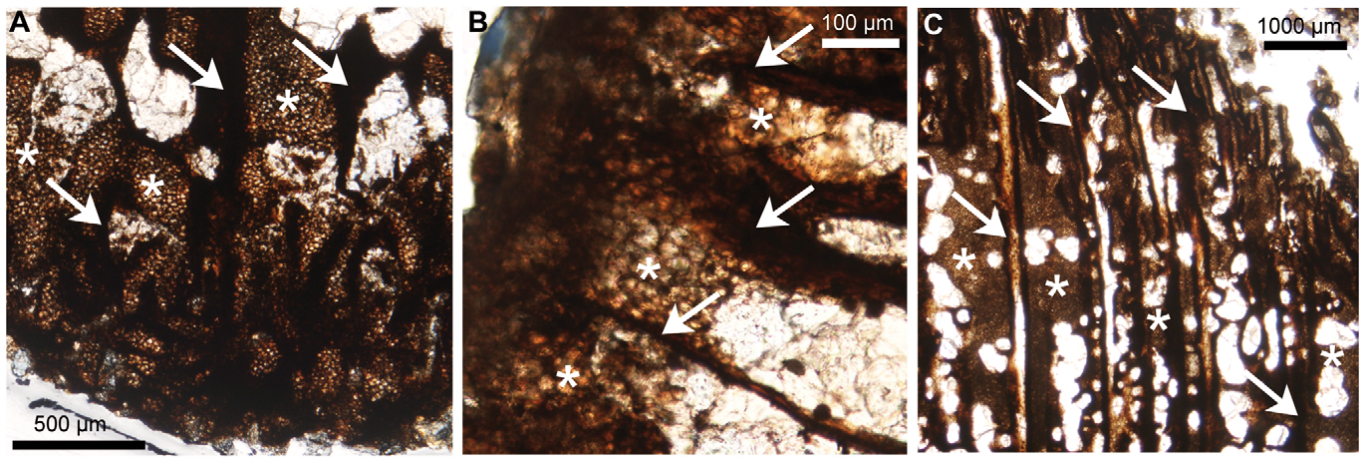
Thin-sections showing remnants of primary cartilage in *Hypacrosaurus*. Longitudinal sections a quadrate (distal end) (**A**), a basisphenoid (**B**) and a tibia (proximal end) (**C**). Primary cartilage is organized into long straight tubes (asterisks), oriented toward the direction of growth, and separated by bone trabeculae (arrows). The junction between these cartilaginous tubes and the bone trabeculae, i.e., the chondro-osseous junction, is undulating (as opposed to straight). Photographs taken under natural light.

As expected, the endochondral bones that were sectioned displayed remnants of primary cartilage on their edges: the basisphenoid, the basioccipital, the supraoccipital and the exoccipital (i.e., chondrocranial bones forming the basicranium), the quadrate, the prootic and the sclerotic (i.e., endochondral bones forming the sensory capsules). No cartilage was found on the laterosphenoid, the orbitosphenoid or the presphenoid; we suggest that this is more a preservation bias rather than an unambiguous absence. The primary cartilage of these specimens is organized into long straight tubes of hypertrophic chondrocytes, separated by bone trabeculae (Figure 3). This tubular arrangement, oriented in one direction is observed in growth cartilages that provide linear growth (i.e., epiphyseal plates of long bones and synchondroses of the cranial base). These cartilaginous tubes give an undulating shape to the junction between bone and calcified cartilage (i.e., the chondro-osseous junction, otherwise straight in mammals and non-avian sauropsids [17], [30]), which is characteristic of the epiphyseal growth plates of birds and non-avian dinosaurs ([17], [30], see also the tibial epiphyseal growth plate of *Hypacrosaurus* in Figure 3C). Secondary cartilage distinctively lacks this linear organization and therefore, primary and secondary cartilage cannot be misidentified. One could argue that the islets in the dentary are nothing more than remnants of Meckel's cartilage, a primary cartilage rod that progressively becomes enveloped by the dentary during development [7]. However, these islets have no topographical relationship with Meckel's cartilage, they are located in the dorsal region of the dentary, while Meckel's cartilage would be located ventrally, as it is in extant amniotes, and they differ histologically from the primary cartilage of Meckel's, that usually stays hyaline in most taxa (i.e., uncalcified, with few and small chondrocytes encased in a very large amount of extracellular matrix) [7]. Moreover, no remnants of Meckel's cartilage were found in the dentary of MOR 548. We suggest that the hyaline nature of Meckel's cartilage did not allow its fossilization.

## Discussion

From a histological perspective, little is known about dinosaur skull development, especially during early ontogenetic phases. Indeed, only four studies on juvenile and adult non-avian dinosaurs describe the histology of cranial bones: the frontoparietals of pachycephalosaurs [31], [32]; the parietals of *Centrosaurus* [33] and *Triceratops* [34], [35]. There are, to our knowledge, no studies on the bone microstructure of earlier ontogenetic stages (e.g., embryos, post- and circum-hatching stages) of dinosaur skulls. This study investigating dinosaurian secondary chondrogenesis presents the youngest non-avian dinosaur skulls ever shown from a histological perspective.

In extant birds, secondary cartilages resist and absorb mechanical stress. Secondary cartilages arise in sites experiencing mechanical stresses (e. g. Figure 1C) such as sutures, articular surfaces (in order to prevent abrasion [20]) and points of insertion of ligaments or masticatory muscles [15]. Likewise, *Hypacrosaurus* displays secondary cartilages at three sites where mechanical factors can be inferred (Figure 1D):

1. On the surangular, the location of the nodule is consistent with preventing abrasion, because it is where it articulates with the quadrate. Moreover, since the section is located in the vicinity of the insertion sites of masticatory muscles (such as *M. depressor mandibulae* and *M. Pterygoideus ventralis* [36]), a muscular induction could also be considered. Similarly, the surangular of birds displays secondary cartilages (Figure 1C, Figure 2C; [18], [20], [21]).
2. The location of the nodule on the maxilla could be puzzling at first; it is not a suture, nor an articular surface, nor has it previously been described as being a muscle insertion site. However, as the nodule directly faces the coronoid process of the dentary we hypothesize that this nodule might have arisen in response to the “pressure” of the coronoid process; or in response to the mechanical stress of a ligamentous or muscular insertion, possibly linking the coronoid process and the maxilla. Further investigation of the histology of a coronoid process in this particular area could shed light on this possibility and be of interest for hadrosaur jaw mechanics.
3. Finally, we hypothesize that the development of secondary cartilaginous islets in the dentary was induced by the mechanical stress of tooth formation and growth. The growth of the dentary is different in non-avian dinosaurs and extant birds because the latter do not possess teeth. Therefore, no direct comparison with extant birds is possible. However, similar secondary cartilage islands are found in mammalian alveolar processes, and their formation allows rapid growth in the mandible [37], [38]. Therefore, we hypothesize that this represents a convergent evolution allowing fast growth in the mandible of *Hypacrosaurus*. This is also supported by the bone microstructure (not only of the dentary, but in the vast majority of the investigated areas, *data not shown*), showing a highly cellular and fibrous primary bone, with numerous and large vascular spaces (e.g., Figure 2A,E); altogether suggesting an extremely high velocity of growth (if not an embryonic potential of growth).

In extant birds, secondary cartilages are found in the cranial vault, in the face, and mostly in the skeleton involved with the masticatory function (Figure 1C and see review [7]). The formation of this skeletal tissue is species-specific and is dictated by the mechanical forces of the mode of feeding [7], [13]. Similarly, in *Hypacrosaurus* we identified secondary cartilage on bones directly involved with the chewing function, the dentary and the surangular in the lower jaw, and the maxilla in the upper jaw (Figure 1D). It is highly probable that more sites were present but were impossible to identify because of poor preservation. It is also possible that additional sites of secondary chondrogenesis existed during the embryonic development of *Hypacrosaurus*, or that new and different nodules arise at later ontogenetic stages, as in birds [20], [21]. Any confusion with primary cartilage of endochondral bones of the skull can be avoided, first because it is not found on the same types of bones, and second, because it is organized differently with straight tubes of cartilage, oriented in one direction and with an undulating chondro-osseous junction. This undulating chondro-osseous junction is also present in the epiphyseal growth cartilages of the long bones of non-avian dinosaurs and birds (Figure 3C; [17], [30]). This is a shared derived anatomical character corroborating the inclusion of birds within the Dinosauria [30]. We describe it here for the first time in the cartilaginous skull of a non-avian dinosaur.

Most importantly, this study indicates that the craniofacial development of birds and at least one clade of dinosaurs, the Ornithischia, is adapted to resist mechanical stress through secondary chondrogenesis.

Induced fracture repair did not produce secondary cartilage in lissamphibians [27] or in lepidosaurians [28], and in crocodilians (the closest living relatives of birds), secondary chondrogenesis was not observed during the development of *Alligator mississippiensis* [29], nor has it been reported during reptilian development [28]. Therefore, because this process fails to occur in any other extant lissamphibian and non-avian sauropsid, we hypothesize that avian and *Hypacrosaurus* secondary cartilages are homologous. If this is the case, and as a result of its inferred presence in their common ancestor secondary cartilage would be present in the other dinosaurian clade, the Saurischia. The alternate hypothesis, that this complex ability of the periosteum to switch from osteogenesis to chondrogenesis evolved independently, seems less plausible. The discovery of “avian” secondary cartilage in a non-avian dinosaur further solidifies the dinosaurian origin of birds and suggests the creation of the more appropriate term “dinosaurian” secondary cartilage. Further histological analyses are underway to study members of the Saurischia, such as non-avian theropod material.

## Materials and Methods

The disarticulated MOR (Museum of the Rockies) 548 specimens were collected from an exceptional hadrosaur nesting ground that has yielded dozens of disarticulated embryos and post hatchlings from at least fifteen individuals of *Hypacrosaurus stebingeri* (Ornithischia, Lambeosaurinae), in the Upper Cretaceous (Campanian) Two Medicine Formation of Montana [6]. The elements used in this study were selected carefully from the collections, in order to represent the approximate same growth stage (i.e., post-hatching, a few months old) with an estimated skull length of 20 cm. A composite skull of a similar size is still on display at the MOR. So far, no texture by which secondary cartilage could be recognized on gross examination was found. In order not to lose any data concerning the size and the morphology of the disarticulated bones, molds and casts were made prior to histological analysis. In total, twenty-five elements were sectioned (Table 1) according to standard fossil thin-sectioning techniques [39]. Specimens were embedded in polyester resin and sectioned with a diamond powder disk on a precision saw. Two to five thin-sections were made of each element, with various cut orientations (i.e., sagittal, parasagittal, transverse and coronal) in order to study multiple potential secondary chondrogenesis sites such as articulations and sutural areas involving membrane bones (Table 2). Sections were then mounted on glass slides, ground and polished. Completed thin section slides were observed with a Nikon Optiphot-Pol polarizing microscope. Photomicrographs were taken with a Nikon DS-Fi1 digital sight camera and the NIS-Elements BR 3.0 software.

**Table 1:** List of the thin-sectioned bones.

Endochondral bones	Membrane bones	
basioccipital*	dentary*	prefrontal
basisphenoid*	frontal	premaxilla
exoccipital*	jugal	quadratojugal
laterosphenoid	lacrimal	squamosal
orbitosphenoid	maxilla*	surangular*
presphenoid	nasal	
prootic*	palatine	
quadrate*	parietal	
sclerotic*	postorbital	
supraoccipital*	predentary	

Bones showing cartilage (remnants of primary cartilage for endochondral bones, and secondary cartilage for membrane bones) are indicated by an asterisk (*).

**Table 2:** List of the articulations studied for the investigation of secondary chondrogenesis.

Articulations	
dentary-predentary	nasal-frontal
dentary-surangular	nasal-prefrontal
frontal-frontal	palatine-maxilla
frontal-nasal	palatine-pterygoid
frontal-parietal	parietal-frontal
frontal-postorbital	parietal-squamosal
jugal-lacrimal	postorbital-frontal
jugal-maxilla	postorbital-parietal
jugal-quadratojugal	prefrontal-nasal
lacrimal-premaxilla	squamosal-parietal
maxilla-jugal	squamosal-postorbital
maxilla-premaxilla	squamosal-quadrate
maxilla-pterygoid	surangular-quadrate*

The elements studied were all disarticulated, but the numerous sections allowed an examination of several sutural edges of a bone (or the inferred areas of contact with other bones). The first-named component indicates the bone that was sectioned. The asterisk (*) indicates where secondary cartilage was found.

## Supporting Information

Text S1 Informations about Mammalian and Teleostean secondary cartilages.
(DOC)
