# Supplementary material for: First Evidence of Dinosaurian Secondary Cartilage in the Post-Hatching Skull of Hypacrosaurus stebingeri (Dinosauria, Ornithischia)
Source: PLoS One. 2012 Apr 30;7(4):e36112. doi: 10.1371/journal.pone.0036112 (PMC3340333; doi:10.1371/journal.pone.0036112)
Supplement: Text S1 — Informations about Mammalian and Teleostean secondary cartilages. (DOC) [file pone.0036112.s001.doc]

**Supporting information**

Mammalian and Teleostean secondary cartilages

Secondary cartilage has only been reported for two species of teleosts: *Poecilia* *sphenops,* on its dentary, maxilla and cleithrum [1], and *Salmo salar* on its dentary [2,3]. However, whether this is mechanically induced and/or maintained is unknown. In mammals, mechanical stimulation is not required for the initiation of secondary cartilages (it is required however for their maintenance). Mammalian secondary cartilages are mostly located in the mandible (in the symphysis, and on the alveolar, condylar, and coronoid processes of the dentary [4-9]; and on the clavicle [10]). However, not all cartilages on the condylar process are secondary. In humans and in rats, the condylar cartilage is secondary, arising from periosteal cells of the condylar process. In mice however, the condylar cartilage is a sesamoid, arising, as does the patella (knee cap) in a separate aggregation of cells beside the condylar process, which subsequently fuses with the condylar process [11].

**References**

1. Benjamin M (1989) The development of hyaline-cell cartilage in the head of the black molly, *Poecilia sphenops*. Evidence for secondary cartilage in a teleost. J Anat 164: 145-155.

2. Witten PE, Hall BK (2002) Differentiation and growth of kype skeletal tissues in anadromous male Atlantic Salmon (*Salmo salar*). Int J Dev Biol 46: 719-730.

3. Gillis JA, Witten PE, Hall BK (2006) Chondroid bone and secondary cartilage contribute to apical dentary growth in juvenile Atlantic salmon *Salmo salar* (Linnaeus (1758). J Fish Biol 68: 1133-1143.

4. Richany SF, Bast TH, Anson BJ (1956) The development of the first branchial arch in man and the fate of Meckels cartilage. Q Bull Northwest Univ Med Sch 30: 331-355.

5. Beresford WA (1981) Chondroid Bone, Secondary Cartilage and Metaplasia. Baltimore : Urban and Schwarzenberg. 454 p.

6. Goret-Niçaise M, Lengelé B, Dhem A (1984) The function of Meckel’s and secondary cartilages in the histomorphogenesis of the cat mandibular symphysis. Arch Anat Microsc Morphol Exp 73: 291-303.

7. Goret-Niçaise M (1986) La croissance de la mandibule humaine: conception actuelle. Thesis University of Louvain (UCL). Bologna: Nauwelaerts.

8. Vinkka-Puhakka H, Thesleff I (1992) Initiation of secondary cartilage in the mandible of the Syrian hamster in the absence of muscle function. Arch Oral Biol 38: 49-54.

9. Bareggi R, Narducci P, Grill V, Sandrucci MA, Bratina F (1994) On the presence of secondary cartilage in the mental symphyseal region of human embryos and fetuses. Surg Radiol Anat 16: 379-84.

10. Tran S, Hall BK (1989) Growth of the clavicle and development of clavicular secondary cartilage in the embryonic mouse. Acta Anat 135: 200-207.

11. Hall BK (2005) Bones and cartilage, Developmental and evolutionary skeletal biology. San Diego: Elsevier. 760 p.
